# Editorial: Analyzing the Relationship Between Dietary Patterns, Health Outcomes, and Individual Food Choices

**DOI:** 10.3389/fnut.2022.840554

**Published:** 2022-02-01

**Authors:** Francesco Visioli, Francesco Sofi

**Affiliations:** ^1^Department of Molecular Medicine, University of Padova, Padua, Italy; ^2^IMDEA-Food, Madrid, Spain; ^3^Department of Experimental and Clinical Medicine, University of Florence, Florence, Italy

**Keywords:** diet, obesity, Mediterranean diet, inflammation, personalized nutrition

Diet is a major contributor to human health and proper food choices can greatly improve prognosis (1). The mechanisms of action underlying the effects of selected food items are still under active investigation. This is why it is important to collect epidemiological, experimental, and biochemical data on individual food choices and health outcomes. One example (but there are many other ones) is that of the Mediterranean diet, where a collection of ample epidemiological evidence (2) coupled with literally hundreds of biochemical studies elucidating the cellular actions of selected components such as olive oil (3) or legumes (4), and at least one large clinical trial, i.e., the PREDIMED (5), built the basis for its election as one of the healthiest diets worldwide.

In this issue of Frontiers in Nutrition, we compiled 22 articles spanning the whole area of human nutrition, from, e.g., restricted time feeding to the metabolic differences between refined and whole grains. Low-grade, chronic inflammation plays an important role in the development or prevention of degenerative diseases such as atherosclerosis, cardiovascular disease, neurodegeneration, and cancer (6). The diet inflammatory index is one way to estimate the link between the pro- or anti-inflammatory potential of the food we eat and the health outcomes associated with it. Future investigations should, indeed, take this and other parameters into account in addition to the mere calorie intake and/or macronutrient profiles.

Thanks to the progresses made by food technology, modern diets are—on average—more nutritious and affordable than the ancient ones. One issue that is gaining traction is that of the so-called ultra-processed foods (7). Even though this notion is highly debated because of the complete lack of biological plausibility (8), data are accumulating that show how (based on current classifications) high consumption of this food category might be associated with poorer prognosis in various areas, including the psychiatric one. This is certainly an issue worth exploring more in depth, leaving ideology aside.

In summary, this Research Topic of Frontiers in Nutrition contributes to increasing our knowledge of how dietary choices affect our health and will help shape informed public health policies. In the future, the use of artificial intelligence, machine learning, and appropriate analysis of big data will further improve our dietary profiles, leading to personalized nutrition based on an individual's genetic background (9). We envision a strong acceleration of nutrigenomics, nutrigenetics, and epigenetics to implement solid guidelines in the developed and developing worlds.

## Author Contributions

FV and FS wrote the article. All authors contributed to the article and approved the submitted version.

## Conflict of Interest

The authors declare that the research was conducted in the absence of any commercial or financial relationships that could be construed as a potential conflict of interest.

## Publisher's Note

All claims expressed in this article are solely those of the authors and do not necessarily represent those of their affiliated organizations, or those of the publisher, the editors and the reviewers. Any product that may be evaluated in this article, or claim that may be made by its manufacturer, is not guaranteed or endorsed by the publisher.
